# The protective role of self-esteem for functional well-being in the context of cancer-related distress among young adults with cancer

**DOI:** 10.1007/s10865-026-00648-4

**Published:** 2026-03-22

**Authors:** Sean McHugh, Katie Darabos, Shannon Desbiens

**Affiliations:** 1 Rutgers School of Public Health, Department of Health Behavior, Society, and Policy, 683 Hoes Lane West, Piscataway, NJ 08854 USA; 2 Rutgers Cancer Institute, 195 Little Albany Street, New Brunswick, NJ 08901 USA

**Keywords:** Cancer, Distress, Functional well-being, Oncology, Self-esteem, Young adults

## Abstract

Functional well-being is an important component of a fulfilled life (e.g., the ability to work and enjoy life). Among young adults (YAs) with cancer, functional well-being may be diminished due to resulting distress from the cancer experience. The moderating role of psychological factors (e.g., self-esteem) in this relationship is poorly understood. To understand the role of self-esteem in the relationship between cancer-related distress and functional well-being among YAs with cancer. 57 YAs with cancer (aged 18–45) diagnosed completed measures self-esteem (Rosenberg Self-Esteem Scale), cancer-related distress (Impact of Events Scale-Revised), and functional well-being (Functional Assessment of Cancer Therapy- General). Significant main effects were observed for cancer-related distress (*β *= – 0.26, *p* < 0.01) and self-esteem (*β* = 0.46, *p* < 0.001) on functional well-being. This was qualified by a significant cancer-related distress x self-esteem interaction (*β* = 0.214, *p* < 0.05). Simple slopes analysis revealed that cancer-related distress and functional well-being was moderated by self-esteem (*β* = – 0.48, *p* < 0.001), such that high levels of cancer-related distress were associated with low levels of functional well-being among YAs with cancer who reported relatively low levels of self-esteem. This relationship was not significant at high levels of self-esteem. Our results underscore the role of self-esteem as a potential psychological resource for YA cancer patients with high levels of cancer-related distress. Targeted interventions aimed at promoting self-esteem may be particularly important for protecting against cancer-related distress and promoting functional well-being in this vulnerable population.

## Background

More than 80,000 young adults (YAs) (aged 18–39) are diagnosed with cancer each year in the United States (Miller et al., 2020). While survival rates for YAs with cancer are relatively high with more than 80% of YAs surviving to at least five years past their diagnosis, cancer during young adulthood is a profoundly distressing experience (Husson & Zebrack, 2017; Miller et al., 2020; Stevens et al., 2024). YAs with cancer experience challenges to achieving normative developmental milestones, such as graduating college, pursuing new job opportunities, forming social and romantic relationships, and starting a family, thereby derailing them from typical YA trajectories (Arnett, 2000; Janssen et al., 2025). Often considered a developmentally “off-time” disease, cancer during young adulthood also exposes YAs with cancer to inherently distressing topics early on, such as financial toxicity (Stevens et al., 2024), infertility (Canzona et al., 2021), and premature fears of mortality, all of which may threaten their emerging sense of autonomy and identity (Nilsson et al., 2020).

In addition, YAs with cancer commonly report long-lasting negative emotional effects (i.e., cancer-related distress), which can lead to increased burden when carrying out daily activities and result in lower satisfaction (i.e., functional well-being). In this context, functional well-being is defined as the extent to which illness affects a person’s ability to fulfill daily roles and responsibilities (e.g., tasks, work, and everyday activities), as well as their ability to engage in and derive enjoyment from everyday life and their perceived satisfaction with their quality of life. Importantly, YAs with cancer often report higher levels of distress than non-cancer peers, as well as older adults with cancer, putting them at heightened risk for adverse effects on functional well-being (Bradford et al., 2022; Burgoyne et al., 2015; Smrke et al., 2020; Wittwer et al., 2023). Aligned with the Conservation of Resources theory (Hobfoll, 1989), as stressors, such as cancer, threaten key resources (e.g., psychological well-being, autonomy, social roles), stress accumulates. Accordingly, the persistent threat to individuals’ psychological, social, and functional resources may increase vulnerability to cancer-related distress.

Experiences of distress after cancer can take a variety of forms. Compounded emotional remnants of the cancer experience coupled with biological and psychosocial risk factors (e.g., young age, advanced cancer stage, poor social support) leave YAs with cancer more prone to experiencing cancer-related distress (Han et al., 2020; Van Noyen et al., 2022; Xiao et al., 2025). The untimeliness of cancer during young adulthood can also reshape identity formation (e.g., pursuing careers, hobbies, and forming social and romantic relationships), a key developmental milestone, and introduce feelings of fear, sadness, uncertainty, anxiety, and depression (Brandenbarg et al., 2019; Jung et al., 2022; Stevens et al., 2024; Walker & Lippard, 2025; Wang & Feng, 2022). Consequently, YAs with cancer report difficulty in their ability to perform instrumental activities of daily living (e.g., aspects of self-care) and social roles (e.g., poor working ability, lower life satisfaction, lower contentment with quality of life) (i.e., functional well-being) (Bradford et al., 2022; Martinez-Calderon et al., 2023; Smrke et al., 2020).

Self-esteem (i.e., feelings of self-confidence, self-worth) has been identified as a valuable psychological resource in cancer patients, often acting as a buffer against psychological distress by boosting internal coping mechanisms (Cohen & Wills, 1985; Niveau et al., 2021; Tsai et al., 2021). Self-esteem plays a major role in young adult development by helping individuals to facilitate emotion regulation and sustain motivation, both of which are crucial to daily functioning (Sanchez-Sanchez et al., 2025; Timko Olson et al., 2024). Consistent with the stress-buffering hypothesis, which suggests that internal (e.g., self-esteem) or external (e.g., social support) resources may mitigate the negative impacts of stress on well-being, self-esteem may serve to protect functional well-being from the negative impacts of cancer-related distress in this population (Cohen & Wills, 1985).

Previous research has examined how psychosocial (e.g., social support, coping) and psychological variables (e.g., anxiety, depression) are associated with functional well-being among YAs with cancer (Arem et al., 2024; Osmani et al., 2023; Zebrack et al., 2014). However, exploration of the contributions of cancer-related distress and intrapersonal characteristics (e.g., self-esteem) on functional well-being is limited. Long-lasting physical and psychological effects associated with cancer diagnosis and treatment (e.g., hair loss, fatigue, sexual dysfunction) (Lau et al., 2024; Walker & Lippard, 2025; Wittwer et al., 2023) can erode YAs self-confidence and self-worth (e.g., self-esteem). This may lead to depleted emotional resources, resulting in distress, and challenging physical ability, resulting in determinants in functioning (Adamkovič et al., 2022; Darabos et al., 2021; Tsai et al., 2021). By examining self-esteem as a moderator, the present study extends prior work by investigating internal resources that may mitigate the negative impacts of cancer-related distress on functional well-being, thereby providing insight into the mechanisms that could support resilience and promote functional well-being in everyday life in this vulnerable population.

With nearly half of all cancer patients experiencing functional challenges, it is critical to identify factors that influence functional well-being to promote adjustment to cancer and improve quality of life for YAs coping with cancer (Mostarac & Brajković, 2022). To address this gap, this study examined relationships between self-esteem, cancer-related distress, and functional well-being in a sample of YAs with cancer. We hypothesized that self-esteem would be positively associated with functional well-being and cancer-related distress would be negatively associated with functional well-being. We further hypothesized that self-esteem would moderate the relationship between cancer-related distress and functional well-being, such that higher levels of cancer-related distress will be associated with lower levels of functional well-being among those with relatively low self-esteem.

## Methods

Participants were 57 young adults with cancer (aged 18–45) asked to participate in a study on communication and support (blinded for review). YAs with cancer were recruited through social media, blog posts, and email blasts from various cancer-related organizations with a sizable YA population (Army of Women, GRYT Health, Lacuna Loft, and Young Survival Coalition), as well as identified by a state cancer registry. Participants were administered a survey link to complete the questionnaire online and were entered into a lottery to receive a gift card. All participants provided informed consent, and all procedures were approved by the university’s Institutional Review Board (2017 − 0807). Participants were on average 35 years old (*M*_*age*_=35.12, SD = 4.78, Range = 24–42). The majority of participants identified as non-Hispanic white (84.2%), female (96.5%), and had breast cancer (77%). The average time since diagnosis was slightly over 2 years (*M* = 26.46, *SD* = 30.0) (see Table 1).

**Table 1:** Descriptive statistics of the study sample (*N* = 57)

Variable	*N* (%)	Range
Age (*M [SD]*)	35.12 (4.78)	18–45
*Gender*		
Female	55 (96.5)	
Male	2 (3.5)	
*Race/ethnicity*		
White	48 (84.2)	
Black/African American	1 (1.8)	
Asian	2 (3.5)	
Hispanic	6 (10.5)	
*Employment*		
Employed	41 (71.9)	
Not Employed	16 (28.1)	
*Treatment Status*		
Active Tx	7 (12.3)	
Completed Tx	50 (87.7)	
*Cancer Type*		
Breast	44 (77.2)	
Not Breast	13 (22.8)	
Months since Dx (*M [SD]*)	26.46 (30.0)	
History of Anxiety (Yes)	23 (49.1)	
History of Depression (Yes)	27 (47.4)	
*Descriptives of Variables (M [SD])*		
Cancer-related Distress	27.4 (16.7)	
Self-esteem	20.2 (4.91)	
Functional Well-Being	17.7 (5.33)	

### Measures

Self-esteem. Self-esteem was measured using the Rosenberg Self-Esteem Scale, which was developed to measure ones positive and negative feelings about themself (e.g., self-worth, self-confidence) (Rosenberg, 1965). The questionnaire consists of items rated on a 4-point Likert-type scale ranging from 1 = ‘Strongly Disagree’ to 4 = ‘Strongly Agree.’ Scores can range from 10 (low level of self-esteem) to 40 (high level of self-esteem). Sample items included “I feel that I have a number of good qualities” and “I feel that I’m a person of worth, at least on an equal plane with others.” A total scale score was calculated as the sum of all items, with higher scores representing higher self-esteem. Cronbach’s alpha in the present study was 0.86.

Cancer-related distress. Cancer-related distress during the past seven days was measured using the 22-item Impact of Events Scale-Revised (IES-R) scale. The IES-R is a validated 22-item measure used to evaluate symptoms of post-traumatic stress disorder (PTSD) (Beck et al., 2008). Participants were asked to answer each question in the context of their cancer diagnosis. Scale anchors range from 0 = ’Not at all’ to 4 = ‘Extremely’. Raw scores were calculated to determine overall symptom endorsement. Sample items included “Any reminder brought back feelings about cancer” and “I was aware that I still had a lot of feelings about cancer, but I don’t want to deal with them. ” A total scale score was calculated by summing all items, with higher scores representing higher cancer-related distress. Cronbach’s alpha in the present study was 0.76.

Functional Well-being. Functional well-being during the past seven days was measured using the 7-item functional well-being subscale of the Functional Assessment of Cancer Therapy- General (FACT-G). The FACT-G is a validated and widely used scale within the cancer context assessing physical, social, emotional, and functional well-being (Cella et al., 1993). Participants answered questions on a 5-point Likert scale. Scale anchors range from 0 = ‘Not at all’ to 5 = ‘Very much’. Sample items include “I am able to work (include work at home)” and “I am able to enjoy life”. Raw scores were calculated, ranging from 0 to 28. Higher scores indicate better functional well-being. Cronbach’s alpha in the present study was 0.88.

### Data analysis

Descriptive statistics and zero-order correlations were conducted for key study variables. Associations among sociodemographic [age (in years), education (less than college vs. college or more), income (less than $75k vs. more than $75k), employment status (employed vs. not employed), ethnicity (Hispanic vs. not)] psychological [history of a depressive (diagnosed with depression vs. not) or anxiety disorder (diagnosed with anxiety vs. not)] and cancer-related variables [time since diagnosis (in months), cancer type (breast vs. not), cancer stage (0-IV), treatment status (active vs. not)] and functional well-being were examined as possible covariates. Significant associations were included as covariates, in addition to key theory-driven variables identified a priori to control for potential confounders.

Multiple linear regression was used to test the study hypotheses. Relevant covariates were included in the first block, in the second block cancer-related distress and self-esteem included in the second block, and the interaction term [Self-esteem x Cancer-related distress] in the third block. Variables were mean-centered to reduce multicollinearity and the interaction terms were analyzed according to Aiken & West (Aiken et al., 1991). Simple slopes for the association between self-esteem and cancer-related distress were tested at low (-1 SD lower than the mean), average (mean), and high (+ 1 SD higher than the mean) levels of self-esteem.

## Results

### Descriptive statistics and identification of covariates

The total mean score for cancer-related distress was 27.4 (*SD* = 16.7) and the total mean score for self-esteem was 20.2 (*SD* = 4.91). The total mean score for functional well-being was 17.7 (*SD* = 5.33). Significant correlations between study variables were observed; cancer-related distress was negatively associated with functional well-being (*r* = – 0.39, *p* < 0.01), and self-esteem was positively associated with functional well-being (*r* = 0.61, *p* < 0.01). Self-esteem and cancer-related distress were not significantly related (*r* = – 0.23, *p* > 0.05). Employment (*r* = – 0.42, *p* < 0.01), history of depression (*r*=0.33, *p* < 0.05), and history of anxiety (*r* = 0.28, *p* < 0.05) were significantly related to functional well-being and therefore included as covariates. Other key study variables identified a priori were included as covariates based on prior research in the field (e.g., age, education, income, time since diagnosis, cancer type, cancer stage, and active treatment status).(Bradford et al., 2022; Zebrack & Isaacson, 2012).

### Hypothesis testing

**Fig. 1:**
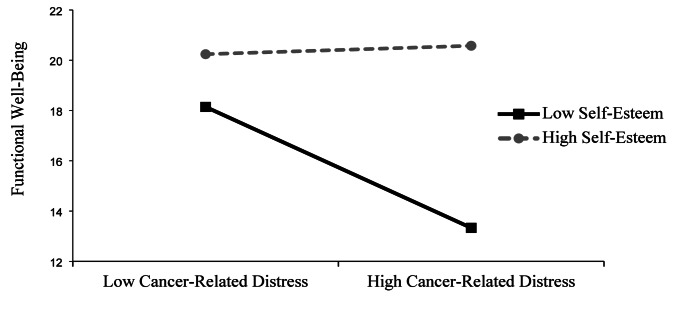
Cancer-related distress and self-esteem on functional well-being. Dashed line indicates nonsignificant simple slope

Regression models revealed that higher levels of self-esteem (*β* = 0.46, *p* < 0.001) were positively associated with functional well-being whereas higher levels of cancer-related distress (*β* = – 0.26, *p* < 0.01) were negatively associated with functional well-being. The overall model was significant, [(*F*(9,47) = 9.97, *p* < 0.001, *R*^*2*^ = 0.66). These main effects were qualified by a significant self-esteem X cancer-related distress interaction (*β* = 0.214, *p* < 0.05), which explained an additional 3% of the variance beyond the main effects (see Table 2). Simple slopes analyses revealed that for YAs with low levels of self-esteem (*β* = – 0.48, *p* < 0.001), high cancer-related distress was associated with lower functional well-being. This relationship was not significant at high levels of self-esteem (see Fig. 1).

**Table 2:** Functional well-being regressed on self-esteem and cancer-related distress

Variable	∆*R*^2^	B	SE	β	LLCI	ULCI
Block 1	0.327**					
Employment		– 3.883**	1.430	– 0.339	– 6.756	– 1.011
Time since dx		0.003	0.021	0.015	– 0.040	0.045
Cancer Type		2.304	1.573	0.182	– 0.855	5.462
Treatment status		– 2.441	1.935	– 0.151	– 6.327	1.445
Hx of depression		2.149	1.584	0.203	– 1.032	5.330
Hx of anxiety		1.640	1.611	0.155	– 1.596	4.876
Block 2	0.296***					
Self-esteem		5.014***	1.027	0.461	2.949	7.079
Cancer-related distress		– 0.085**	0.030	– 0.264	– 0.146	– 0.023
Block 3	0.033*					
Self-Esteem X Cancer-related distress		0.133*	0.062	0.214	0.007	0.258

Regression coefficients reflect values at the end of block 3, with all variables entered into the model. Dx, diagnosis; Hx, history; *SE*, standard error; *LLCI*: lower level 95% confidence interval *(CI)*; *ULCI*: upper level 95% CI; *p* < 0.05*, *p* < 0.01**, *p* < 0.001***

## Discussion

This study aimed to understand the moderating role of self-esteem on the relationship between cancer-related distress and functional well-being among young adults with cancer. Overall, the results provide some support suggesting that the combination of high cancer-related distress and low self-esteem among YA cancer survivors is associated with lower functional well-being. This pattern of interaction was not significant at high levels of self-esteem, potentially suggesting that high levels of self-esteem may be protective in this context. These observations might reflect that high levels of self-esteem are possible when cancer-related distress is high. Accordingly, aligned with the stress buffering hypothesis (Cohen & Wills, 1985), our results suggest that YAs with cancer with high self-esteem may possess greater resources to cope with cancer-related distress, enabling them to maintain a sense of purpose and competence in work and daily tasks.

Considering the potential influence of the largely homogenous population in this study is crucial to understanding these results. Our study consisted primarily of women with breast cancer who were off treatment at the time of enrollment. Certain self-esteem and distress-related concerns among women with breast cancer may be more pronounced due to body image changes caused by cancer-related treatments [e.g., loss of breast(s), scars]. However, other cancer diagnoses and treatments may additionally result in negative impacts to self-esteem and cancer-related distress. For example, alterations in physical appearance, including scars from surgeries and central lines, weight changes, hair loss, loss of a limb, and restricted cognitive and physical functioning are often reported (Schruff et al., 2025; Zeltzer, 1993).

In addition, majority of YAs have already completed active cancer treatment. Therefore, it is possible that many individuals were in a phase of adjustment in which self-esteem may not have been impacted. However, our results are consistent with previous research finding moderate levels of self-esteem among this population (Wurz & Brunet, 2020). Penn et al. (2017) suggested that despite threats to self-esteem associated with cancer and its treatment, some YA cancer survivors are not as impacted following cancer. Thus, it becomes important to identify YAs with low self-esteem and potential co-occurring factors that in combination with low self-esteem, are associated with decreases in well-being. In the present sample, 17% of YAs reported low levels of self-esteem, defined as scores below 15 on the Rosenberg Self-Esteem Scale.

Understanding internal and external factors that aid self-esteem as a protective resource may be important for this population. For example, one study highlights the impact of social support networks on levels of self-esteem in adolescents and young adults with cancer, showing that higher perceived social support is related to higher self-esteem (Walker & Lippard, 2025). Thus, examining the social support networks of YAs with cancer may provide insight into both increased risk for poor functional well-being as well as pathways for targeting self-esteem to protect against the negative effects of cancer-related distress.

Targeting self-esteem may offer protective benefits against the adverse effects of cancer-related distress on functional well-being. While current screening tools in psychosocial oncology clinics (e.g., the Distress Thermometer and the Hospital Anxiety and Depression Scale) focus on distress as a broader concept of the cancer experience (Fitch et al., 2024; Zebrack et al., 2014), self-esteem is often minimally addressed. As research suggests, prolonged distress from the unique developmental challenges faced by YAs during their cancer experience can significantly impact well-being (Janssen et al., 2025; Timko Olson et al., 2024). Addressing self-esteem in this context may be multifaceted and necessitates an approach towards psychoeducation around active coping skills (e.g., positive reappraisal, acceptance, emotional support) as critical tools for moving forward and gaining a sense of meaning back to promote life satisfaction and feelings of fulfillment for carrying out daily tasks (Adamkovič et al., 2022; Inci et al., 2021). Exploring individualized facets of self-esteem (e.g., self-confidence, self-worth, identity) may also provide valuable insights for the development of more tailored interventions focused on promoting aspects of self-esteem that may buffer cancer-related distress and boost functional well-being in YAs with cancer. For example, interventions for young adults experiencing visible treatment-related changes (e.g., hair loss, loss of breast(s), or scarring) may focus on body image and self-worth through cognitive restructuring and self-compassion exercises (Almeida et al., 2025; Zhao et al., 2023). Future studies should explore whether self-esteem has long-term implications for protecting YAs with cancer from cancer-related distress in the context of functional well-being.

## Implications

Positive feelings of self-confidence and self-worth, as well as a secure identity (i.e., self-esteem) seemed to buffer the effects of cancer-related distress on functional well-being. While our sample population reported relatively low levels of cancer-related distress and relatively high levels of functional well-being, these findings have implications for future development of standardized assessments and targeted interventions for YAs with cancer. However, more work is needed to address whether self-esteem has a similar effect on the relationship between cancer-related distress and functional well-being longitudinally.

## Limitations

Our study is not without limitations. Firstly, this study was completed in a relatively small sample size. In the future, studies should look at these relationships within a larger population of YAs with cancer. This study used self-report measures to assess self-esteem, cancer-related distress, and functional well-being. Future research should consider implementing multimethod approaches to reduce potential self-report bias. Additionally, this was a cross-sectional design. Future studies would benefit from examining self-esteem in a longitudinal context as it relates to cancer-related distress and functional well-being. This might be particularly important in the clinical setting for understanding how these relationships unfold over time. As mentioned prior, most of the study participants were white, non-Hispanic, female, and diagnosed with breast cancer, which may impact the generalizability of our findings. Other cancer types, as well as belonging to historically underrepresented groups (e.g., racial and ethnic minorities, individuals of lower socioeconomic status, and gender minorities), may shape cancer experiences in ways that have differing implications for self-esteem and levels of distress. Future research should explore these relationships within a broader population of YAs with cancer during varying levels of distress.

## Conclusion

The findings of our study are impactful for YAs with cancer experiencing low self-esteem. Our results underscore the importance of self-esteem in protecting against cancer-related distress which has implications for detriments in functional well-being. Self-esteem, which encompasses high levels of self-confidence and worth, may be critical for YAs with cancer engaging in meaningful work and feeling satisfied with daily life, and may be advantageous in providing effective strategies for YAs with cancer from diagnosis and into survivorship to promote a better quality of life.

## Data Availability

The data that support the findings of this study are available from the corresponding author upon reasonable request.

## References

[CR1] Adamkovič, M., Fedáková, D., Kentoš, M., Bozogáňová, M., Havrillová, D., Baník, G., Dědová, M., & Piterová, I. (2022). Relationships between satisfaction with life, posttraumatic growth, coping strategies, and resilience in cancer survivors: A network analysis approach. *Psychooncology*, *31*(11), 1913–1921. 10.1002/pon.5948 35524705 10.1002/pon.5948 PMC9790334

[CR2] Aiken, L. S., West, S. G., & Reno, R. R. (1991). *Multiple regression: Testing and interpreting interactions*. Sage.

[CR3] Almeida, M., Griff, M. I., & Brandão, T. (2025). Coping and positive body image in young women with breast cancer: The buffering role of social support. *Healthcare (Basel)*, *13*(3). 10.3390/healthcare13030346 10.3390/healthcare13030346 PMC11817120 39942534

[CR4] Arem, H., Duarte, D. A., White, B., Vinson, K., Hinds, P., Ball, N., Dennis, K., McCready, D. M., Cafferty, L. A., & Berg, C. J. (2024). Young adult cancer survivors’ perspectives on cancer’s impact on different life areas post-treatment: A qualitative study. *Journal of Adolescent and Young Adult Oncology*, *13*(5), 748–759. 10.1089/jayao.2024.0021 38695773 10.1089/jayao.2024.0021 PMC11564678

[CR5] Arnett, J. J. (2000). Emerging adulthood. A theory of development from the late teens through the twenties. *American Psychologist*, *55*(5), 469–480. 10842426

[CR6] Beck, J. G., Grant, D. M., Read, J. P., Clapp, J. D., Coffey, S. F., Miller, L. M., & Palyo, S. A. (2008). The impact of event scale-revised: Psychometric properties in a sample of motor vehicle accident survivors. *Journal Of Anxiety Disorders*, *22*(2), 187–198. 10.1016/j.janxdis.2007.02.007 17369016 10.1016/j.janxdis.2007.02.007 PMC2259224

[CR7] Bradford, N. K., McDonald, F. E. J., Bibby, H., Kok, C., & Patterson, P. (2022). Psychological, functional and social outcomes in adolescent and young adult cancer survivors over time: A systematic review of longitudinal studies. *Psycho-Oncology*, *31*(9), 1448–1458. 10.1002/pon.5987 35734846 10.1002/pon.5987 PMC9544373

[CR8] Brandenbarg, D., Maass, S. W. M. C., Geerse, O. P., Stegmann, M. E., Handberg, C., Schroevers, M. J., & Duijts, S. F. A. (2019). A systematic review on the prevalence of symptoms of depression, anxiety and distress in long-term cancer survivors: Implications for primary care. *European Journal of Cancer Care*, *28*(3), e13086. 10.1111/ecc.13086 31087398 10.1111/ecc.13086 PMC9286037

[CR9] Burgoyne, M. J., Bingen, K., Leuck, J., Dasgupta, M., Ryan, P., & Hoffmann, R. G. (2015). Cancer-related distress in young adults compared to middle-aged and senior adults. *Journal of Adolescent and Young Adult Oncology*, *4*(2), 56–63. 10.1089/jayao.2014.0005. 26812552 10.1089/jayao.2014.0005

[CR10] Canzona, M. R., Victorson, D. E., Murphy, K., Clayman, M. L., Patel, B., Puccinelli-Ortega, N., McLean, T. W., Harry, O., Little-Greene, D., & Salsman, J. M. (2021). A conceptual model of fertility concerns among adolescents and young adults with cancer. *Psychooncology*, *30*(8), 1383–1392. 10.1002/pon.5695 33843104 10.1002/pon.5695 PMC8363581

[CR11] Cella, D. F., Tulsky, D. S., Gray, G., Sarafian, B., Linn, E., Bonomi, A., Silberman, M., Yellen, S. B., Winicour, P., Brannon, J., et al. (1993). The functional assessment of cancer therapy scale: Development and validation of the general measure. *Journal of Clinical Oncology*, *11*(3), 570–579. 10.1200/jco.1993.11.3.570 8445433 10.1200/JCO.1993.11.3.570

[CR12] Cohen, S., & Wills, T. A. (1985). *Stress, social support, and the buffering hypothesis*. American Psychological Association. 10.1037/0033-2909.98.2.310] 3901065

[CR13] Darabos, K., Renna, M. E., Wang, A. W., Zimmermann, C. F., & Hoyt, M. A. (2021). Emotional approach coping among young adults with cancer: Relationships with psychological distress, posttraumatic growth, and resilience. *Psychooncology*, *30*(5), 728–735. 10.1002/pon.5621 33368816 10.1002/pon.5621 PMC10865384

[CR14] Fitch, M. I., Nicoll, I., & Burlein-Hall, S. (2024). Screening for psychosocial distress: A brief review with implications for oncology nursing. *Healthcare (Basel)*. 10.3390/healthcare12212167 10.3390/healthcare12212167 PMC11545043 39517379

[CR15] Han, C. J., Gigic, B., Schneider, M., Kulu, Y., Peoples, A. R., Ose, J., Kölsch, T., Jacobsen, P. B., Colditz, G. A., Figueiredo, J. C., Grady, W. M., Li, C. I., Shibata, D., Siegel, E. M., Toriola, A. T., Ulrich, A. B., Syrjala, K. L., & Ulrich, C. M. (2020). Risk factors for cancer-related distress in colorectal cancer survivors: One year post surgery. *Journal of Cancer Survivorship*, *14*(3), 305–315. 10.1007/s11764-019-00845-y 32166576 10.1007/s11764-019-00845-y PMC7261242

[CR16] Hobfoll, S. E. (1989). Conservation of resources: A new attempt at conceptualizing stress. *American Psychologist*, *44*(3), 513–524. 10.1037/0003-066X.44.3.513 2648906 10.1037//0003-066x.44.3.513

[CR17] Husson, O., & Zebrack, B. J. (2017). Perceived impact of cancer among adolescents and young adults: Relationship with health-related quality of life and distress. *Psycho-Oncology*, *26*(9), 1307–1315. 10.1002/pon.4300 27862627 10.1002/pon.4300

[CR18] Inci, H., Inci, F., Ersoy, S., Karatas, F., & Adahan, D. (2021). Self-esteem, metacognition, and coping strategies in cancer patients: A case-control study. *Journal of Cancer Research and Therapeutics*, *17*(4), 956–962. 10.4103/jcrt.JCRT_618_19 34528548 10.4103/jcrt.JCRT_618_19

[CR19] Janssen, S. H. M., Vlooswijk, C., Bijlsma, R. M., Kaal, S. E. J., Kerst, J. M., Tromp, J. M., Bos, M. E. M. M., van der Hulle, T., Lalisang, R. I., Nuver, J., Kouwenhoven, M. C. M., van der Graaf, W. T. A., & Husson, O. (2025). Health-related quality of life of long-term adolescent and young adult (AYA) cancer survivors compared to a matched normative population: Results of the SURVAYA study. *Journal of Cancer Survivorship*. 10.1007/s11764-025-01818-040528140 10.1007/s11764-025-01818-0

[CR20] Jung, A., Crandell, J. L., Nielsen, M. E., Smith, S. K., Bryant, A. L., & Mayer, D. K. (2022). Relationships among uncertainty, post-traumatic stress disorder symptoms, and quality of life in non-muscle-invasive bladder cancer survivors. *Supportive Care in Cancer*, *30*(7), 6175–6185. 10.1007/s00520-022-07034-1 35437672 10.1007/s00520-022-07034-1

[CR21] Lau, N., Steineck, A., Walsh, C., Fladeboe, K. M., Yi-Frazier, J. P., Rosenberg, A. R., & Barton, K. (2024). Social support resources in adolescents and young adults with advanced cancer: A qualitative analysis. *BMC Palliative Care*, *23*(1), 193. 10.1186/s12904-024-01527-y 39085897 10.1186/s12904-024-01527-y PMC11290203

[CR22] Martinez-Calderon, J., García-Muñoz, C., Heredia-Rizo, A. M., & Cano-García, F. J. (2023). Meaning and purpose in life, happiness, and life satisfaction in cancer: Systematic review with meta-analysis. *Psycho-Oncology*, *32*(6), 846–861. 10.1002/pon.6135 37095608 10.1002/pon.6135

[CR23] Miller, K. D., Fidler-Benaoudia, M., Keegan, T. H., Hipp, H. S., Jemal, A., & Siegel, R. L. (2020). Cancer statistics for adolescents and young adults, 2020. *CA: A Cancer Journal For Clinicians*, *70*(6), 443–459. 10.3322/caac.21637 10.3322/caac.21637 32940362

[CR24] Mostarac, I., & Brajković, L. (2022). Life after facing cancer: Posttraumatic growth, meaning in life and life satisfaction. *Journal of Clinical Psychology in Medical Settings*, *29*(1), 92–102. 10.1007/s10880-021-09786-0 34008123 10.1007/s10880-021-09786-0

[CR25] Nilsson, S., Hård af Segerstad, Y., & Olsson, M. (2020). Worrying about death: An initial analysis of young adult cancer patients’ needs. *Journal of Adolescent and Young Adult Oncology*, *10*(1), 105–108. 10.1089/jayao.2020.0033 32552246 10.1089/jayao.2020.0033

[CR26] Niveau, N., New, B., & Beaudoin, M. (2021). How should self-esteem be considered in cancer patients? *Frontiers in Psychology*, *12*, 763900. 10.3389/fpsyg.2021.763900 34777169 10.3389/fpsyg.2021.763900 PMC8581674

[CR27] Osmani, V., Hörner, L., Klug, S. J., & Tanaka, L. F. (2023). Prevalence and risk of psychological distress, anxiety and depression in adolescent and young adult (AYA) cancer survivors: A systematic review and meta-analysis. *Cancer Medicine*, *12*(17), 18354–18367. 10.1002/cam4.6435 37559504 10.1002/cam4.6435 PMC10523984

[CR28] Penn, A., Kuperberg, A., & Zebrack, B. (2017). Psychosocial issues in adolescent and young adult patients and survivors.

[CR30] Rosenberg, M. (1965). *Society and the adolescent self-image*. Princeton University Press. 10.1515/9781400876136

[CR31] Sanchez-Sanchez, H., Schoeps, K., & Montoya-Castilla, I. (2025). Emotion regulation strategies and psychological well-being in emerging adulthood: Mediating role of optimism and self-esteem in a university student sample. *Behavioral Sciences*, *15*(7), 929. https://www.mdpi.com/2076-328X/15/7/929 40723713 10.3390/bs15070929 PMC12292161

[CR32] Schruff, M. A., Sharp, H., Heidelberg, K. M., R. E., & Daniels, S. (2025). Self-image among adolescent and young adult cancer survivors: A qualitative study. *Journal of Pediatric Psychology*. 10.1093/jpepsy/jsaf085 40985455

[CR33] Smrke, A., Leung, B., Srikanthan, A., McDonald, M., Bates, A., & Ho, C. (2020). Distinct features of psychosocial distress of adolescents and young adults with cancer compared to adults at diagnosis: Patient-reported domains of concern. *Journal of Adolescent and Young Adult Oncology*, *9*(4), 540–545. 10.1089/jayao.2019.0157 32255694 10.1089/jayao.2019.0157

[CR34] Stevens, J. M., Montgomery, K., Miller, M., Saeidzadeh, S., & Kwekkeboom, K. L. (2024). Common patient-reported sources of cancer-related distress in adults with cancer: A systematic review. *Cancer Medicine*, *13*(13), e7450. 10.1002/cam4.7450 38989923 10.1002/cam4.7450 PMC11238242

[CR35] Timko Olson, E. R., Olson, A., Driscoll, M., & Bliss, D. Z. (2024). Psychosocial factors affecting wellbeing and sources of support of young adult cancer survivors: A scoping review. *Nursing Reports*, *14*(4), 4006–4021. https://www.mdpi.com/2039-4403/14/4/293 10.3390/nursrep14040293 PMC11677591 39728654

[CR36] Tsai, P. L., Kuo, T. T., Ku, C. H., Liao, G. S., Lin, C. K., & Pan, H. H. (2021). Self-esteem as a predictor of mental adjustment in patients with breast cancer. *International Journal of Environmental Research and Public Health*, *18*(23), 12588. https://www.mdpi.com/1660-4601/18/23/12588 34886314 10.3390/ijerph182312588 PMC8656551

[CR37] Van Noyen, L., Markovitz, S., Broers, N. J., & Peters, M. L. (2022). Prevalence and predictors of psychological distress in women diagnosed with breast cancer and women without breast cancer: A prospective study of psychological risk and resilience factors. *Journal of Psychosocial Oncology Research and Practice*. https://journals.lww.com/jporp/fulltext/2022/12000/prevalence_and_predictors_of_psychological.1.aspx

[CR38] Walker, K., & Lippard, C. (2025). Exploring the relationship between social support and self-esteem for adolescent cancer survivors. *Cancer Survivorship Research & Care*, *3*(1), 2527027. 10.1080/28352610.2025.2527027

[CR39] Wang, Y., & Feng, W. (2022). Cancer-related psychosocial challenges. *General Psychiatry*, *35*(5), e100871. 10.1136/gpsych-2022-100871 36311374 10.1136/gpsych-2022-100871 PMC9540834

[CR40] Wittwer, A., Sponholz, K., Frietsch, J. J., Linke, P., Kropp, P., Hochhaus, A., & Hilgendorf, I. (2023). Psychosocial distress in young adults surviving hematological malignancies: A pilot study. *Journal of Cancer Research and Clinical Oncology*, *149*(9), 5655–5663. 10.1007/s00432-022-04527-8 36527483 10.1007/s00432-022-04527-8 PMC10356626

[CR41] Wurz, A., & Brunet, J. (2020). Describing and exploring self-esteem, physical self-perceptions, physical activity and self-efficacy in adolescent and young adult cancer survivors. *European Journal of Cancer Care(England)*, *29*(1), e13179. 10.1111/ecc.13179 31647149 10.1111/ecc.13179

[CR42] Xiao, Y., Yang, K., Zhang, F., Li, Z., & Jiang, X. (2025). Post-traumatic stress disorder symptoms and associated factors in newly diagnosed breast cancer survivors: A cross-sectional study. *European Journal of Oncology Nursing: The Official Journal of European Oncology Nursing Society*, *77*, 102776. 10.1016/j.ejon.2024.102776 39828485 10.1016/j.ejon.2024.102776

[CR43] Zebrack, B., & Isaacson, S. (2012). Psychosocial care of adolescent and young adult patients with cancer and survivors. *Journal of Clinical Oncology*, *30*(11), 1221–1226. 10.1200/jco.2011.39.5467 22412147 10.1200/JCO.2011.39.5467

[CR44] Zebrack, B. J., Corbett, V., Embry, L., Aguilar, C., Meeske, K. A., Hayes-Lattin, B., Block, R., Zeman, D. T., & Cole, S. (2014). Psychological distress and unsatisfied need for psychosocial support in adolescent and young adult cancer patients during the first year following diagnosis. *Psychooncology*, *23*(11), 1267–1275. 10.1002/pon.3533 24664958 10.1002/pon.3533

[CR45] Zeltzer, L. K. (1993). Cancer in adolescents and young adults psychosocial aspects. Long-term survivors. *Cancer*, *71*(10 Suppl), 3463–3468. 10.1002/1097-0142(19930515)71:10+<3463::aid-cncr2820711753>3.0.co;2-b 8490896 10.1002/1097-0142(19930515)71:10+<3463::aid-cncr2820711753>3.0.co;2-b

[CR46] Zhao, W., Chong, Y. Y., & Chien, W. T. (2023). Effectiveness of cognitive-based interventions for improving body image of patients having breast cancer: A systematic review and meta-analysis. *Asia-Pacific Journal of Oncology Nursing*, *10*(4), 100213. 10.1016/j.apjon.2023.100213 37089782 10.1016/j.apjon.2023.100213 PMC10120298

